# Shadow-Assisted Sidewall Emission for Achieving Submicron Linewidth Light Source by Using Normal UV Photolithography

**DOI:** 10.1007/s40820-025-01737-w

**Published:** 2025-04-22

**Authors:** Junlong Li, Yanmin Guo, Kun Wang, Wei Huang, Hao Su, Wenhao Li, Xiongtu Zhou, Yongai Zhang, Tailiang Guo, Chaoxing Wu

**Affiliations:** 1 College of Physics and Information Engineering, Fuzhou University, Fuzhou, 350108 People’s Republic of China; 2 Fujian Science & Technology Innovation Laboratory for Optoelectronic Information of China, Fuzhou, 350108 People’s Republic of China

**Keywords:** Submicron light source, Quantum dot, Photoluminescence, Photolithography, Shadow-assisted sidewall emission

## Abstract

Micro light sources are crucial tools for studying the interactions between light and matter at the micro/nanoscale, encompassing diverse applications across multiple disciplines. Despite numerous studies on reducing the size of micro light sources and enhancing optical resolution, the efficient and simple fabrication of ultra-high-resolution micro light sources remains challenging due to its reliance on precise micro-nano processing technology and advanced processing equipment. In this study, a simple approach for the efficient fabrication of submicron light sources is proposed, namely shadow-assisted sidewall emission (SASE) technology. The SASE utilizes the widely adopted UV photolithography process, employing metal shadow modulation to precisely control the emission of light from polymer sidewalls, thereby obtaining photoluminescent light sources with submicron line widths. The SASE eliminates the need for complex and cumbersome manufacturing procedures. The effects of process parameters, including exposure dose, development time, and metal film thickness, on the linewidth of sources are investigated on detail. It is successfully demonstrated red, green, and blue submicron light sources. Finally, their potential application in the field of optical anti-counterfeiting is also demonstrated. We believe that the SASE proposed in this work provides a novel approach for the preparation and application of micro light sources.

**Supplementary Information:**

The online version contains supplementary material available at 10.1007/s40820-025-01737-w.

## Highlights

- The submicron light sources are realized only by the normal UV photolithography process, enabling the realization of submicron light sources with arbitrary patterns.
- A novel and efficient method for the fabrication of submicron light sources is proposed, termed shadow-assisted sidewall emission.
- The submicron light source fabricated by the shadow-assisted sidewall emission exhibits strong scalability and has been proved to be applicable in optical anti-counterfeiting.

**Supplementary Information:**

The online version contains supplementary material available at 10.1007/s40820-025-01737-w.

## Graphical abstract

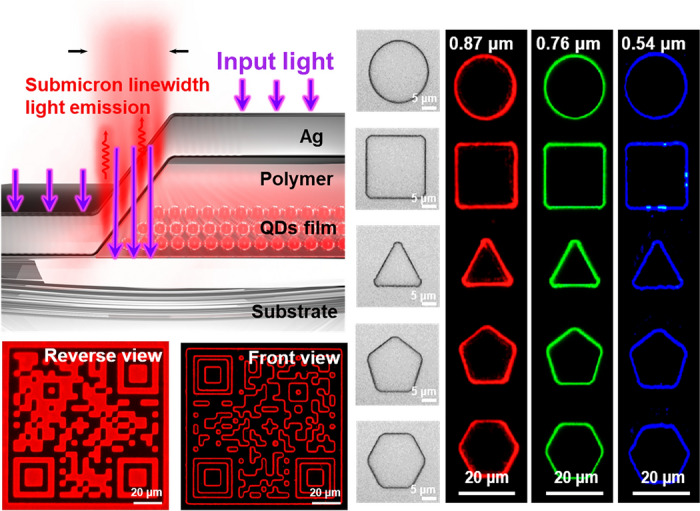

## Introduction

The development and application of microscale light sources have provided unprecedented optical resolution and precision for scientific research, enabling the manipulation of light–matter interactions at the nanoscale [1–3]. This progress not only extends the frontier of optical field control but also establishes a foundation for ultra-high-resolution optical detection, precise light modulation, and the development of novel optical functional devices. With continuous improvements in fabrication techniques, microscale light sources are also evolving toward stability, and tunability, offering new avenues for surpassing the performance limitations of conventional optical devices. The core characteristic of microscale light sources is their ability to generate light output within micron and even submicron spatial ranges, allowing precise control over the interaction region between light and matter, thereby obtaining material information at submicron scales [4–6]. This high-precision control capability is great significance in various fields, particularly in bioimaging, optogenetics, microspectroscopy, and biomedical applications [7–10]. For instance, in bioimaging, high-resolution microscale light sources enhance super-resolution fluorescence microscopy. Wijesooriya reported that photoactivatable BODIPY probe is used as microscale light sources for single-molecule localization-based super-resolution microscopy. It has been proved to be effective for live-cell imaging and is compatible with various biological samples [11]. In optogenetics, they enable precise stimulation of specific cells, thereby enhancing spatial selectivity and experimental controllability. Dai proposed a new type of tiny multimaterial glass fiber, which allows for simultaneous deep neural stimulation and detection for more than 2 weeks at a single cellular level. The microscale light sources promote the development of neuroscience and brain science through the ability to manipulate neural circuits in the deep brain [12]. Therefore, enhancing the optical resolution of microscale light sources to precisely target smaller spatial scales has always been a major objective in scientific research [13–17].

Micro-LEDs, nano-LEDs, quantum dots (QDs), nanowires, micro-semiconductor lasers, and surface-localized plasmon technology are commonly employed for the fabrication of miniature light sources [18–22]. These light sources possess ultra-high-resolution characteristics that enable operation within the micro/nanometer scale range and exhibit exceptional emission performance. Despite the remarkable optoelectronic properties and potential for high levels of integration, their manufacturing processes are intricate and rely on advanced complex fabrication techniques (Fig. S1) [23–26]. Taking Micro-LEDs as an example, it is an important solution for achieving ultra-high-resolution microscale light sources. Typically, photolithography is used to create patterned masks, followed by inductively coupled plasma etching of LED epitaxial wafers to fabricate ultra-high-resolution Micro-LED arrays [27–29]. It has been successful fabrications of Micro-LED arrays with a pitch of 0.4 μm [30]. Nevertheless, these ultra-high-resolution microscale light sources rely on advanced ultraviolet (UV) exposure systems or even electron beam lithography [31]. On the other hand, enhancing the resolution of devices by coupling with precise optical components, such as optical waveguide arrays and microlens arrays, also appears to be a feasible approach. For instance, Micro-LEDs coupled with optical waveguide arrays onto neural probes can significantly improve the spatial resolution of these probes [32, 33]. However, the fabrication of precise optical components still involves various micro-nano fabrication techniques, such as reactive ion etching, chemical vapor deposition, and physical vapor deposition. The manufacturing process is complex and challenging. Therefore, developing a simple and efficient fabrication process for microscale light sources, particularly submicron light sources, is essential for their large-scale application.

In this work, an extremely simple method is proposed, termed shadow-assisted sidewall emission (SASE), which enables the fabrication of submicron light sources only using conventional UV photolithography process. By leveraging the sidewall effect generated during the photolithography process, a QDs-based submicron light source with an ultra-high resolution and a minimum linewidth of ~ 470 nm is fabricated. The submicron light source features a simple structure and an easy fabrication process, avoiding the complexity of traditional micro-nanofabrication techniques. Factors influencing the submicron light source, such as exposure dose during photolithography, development time, and the thickness of the Ag film are investigated. An in-depth analysis of the mechanisms behind the generation of the submicron light source is conducted. It is successfully fabricated red, green, and blue submicron light sources. During the fabrication process, it is found that the interesting method can be applied in optical anti-counterfeiting. Therefore, we demonstrate the fabrication of flexible submicron light sources on a flexible substrate using the SASE method and effectively applied them in optical anti-counterfeiting. Based on the results of this study, we believe that the proposed SASE is a viable method for achieving ultra-high-resolution submicron light sources.

## Experimental Section

### Materials

Photosensitive polymer SPR955, RDP-8003, AZ P4210, AZ GXR 601, and developer solution ZX 238 are purchased from Xi’an Boyan Micro-nano International Technology Co., Ltd. The QDs for red, green, and blue are cadmium selenide core/shell colloidal quantum dots purchased from Suzhou Xingshuo Nanotech Co., Ltd. Ag (99.99%) evaporation material is purchased from ZhongNuo Advanced Material (Beijing) Technology Co., Ltd. The PEN and glass substrate are purchased from South China Science and Technology Co., Ltd.

### Fabrication of the Device

The device is fabricated on a clean glass substrate. First, the glass is cleaned in an ultrasonic bath using acetone, ethyl alcohol, and deionized water for 10 min for each and is blown dry with a nitrogen gun. The CdSe/ZnS QD solution (25 mg mL^−1^) is spin-coated onto the glass substrate at a speed of 500 rpm for 5 s and then 3000 rpm for 40 s, and then it is baked at 120 °C for 10 min. Subsequently, the photosensitive polymer is spin-coated on the QDs layer at 500 rpm for 5 s and then 4000 rpm for 30 s. After soft baking at 90 °C for 90 s, the photosensitive polymer is exposed to UV light through the photomask with patterns for ring openings by using a high-precision ultraviolet lithography machine (MABA6 Gen4). In the lithography process, vacuum contact exposure method is adopted, which can create a vacuum between the mask and the substrate, ensuring intimate contact and enabling high-precision patterning. Subsequently, the ultraviolet light with a wavelength of 365 nm is employed to expose the photosensitive polymer, inducing localized chemical reactions within the polymer material. After exposure, the substrate is immersed in ZX238 developer for 75 s, followed by rinsing with deionized water to eliminate residual developer. The substrate is subsequently dried using nitrogen gas and post-baked at 120 °C for 90 s. Finally, the Ag film is deposited on the patterned substrate via thermal evaporation in a high-vacuum evaporation chamber with a pressure of 2 × 10^–4^ Pa. As for the flexible device, the fabrication procedure follows the same fabrication scheme as the patterned glass substrate by photolithography, except the glass substrate is changed to the flexible PEN substrate.

### Characterization

The morphology and height profile of the fine-patterned device are obtained using a 3D laser microscope (OLYMPUS, OLS4100). Scanning electron microscope (SEM, FEI Helios 450S dual beam FIB) images are used to characterize a cross section of the device. Prior to acquiring the cross-sectional SEM images of the device, a platinum layer is deposited on its surface using ion sputtering. The fluorescence microscopic images of the patterned QD film are obtained by fluorescence microscopy (Olympus BX51M). Photoluminescence (PL) spectra, time-resolved photoluminescence (TrPL), and photoluminescence quantum yield (PLQY) of QDs are characterized using Edinburgh Instruments FLS1000.

## Result and Discussion

### Design of SASE Device

**Fig. 1:**
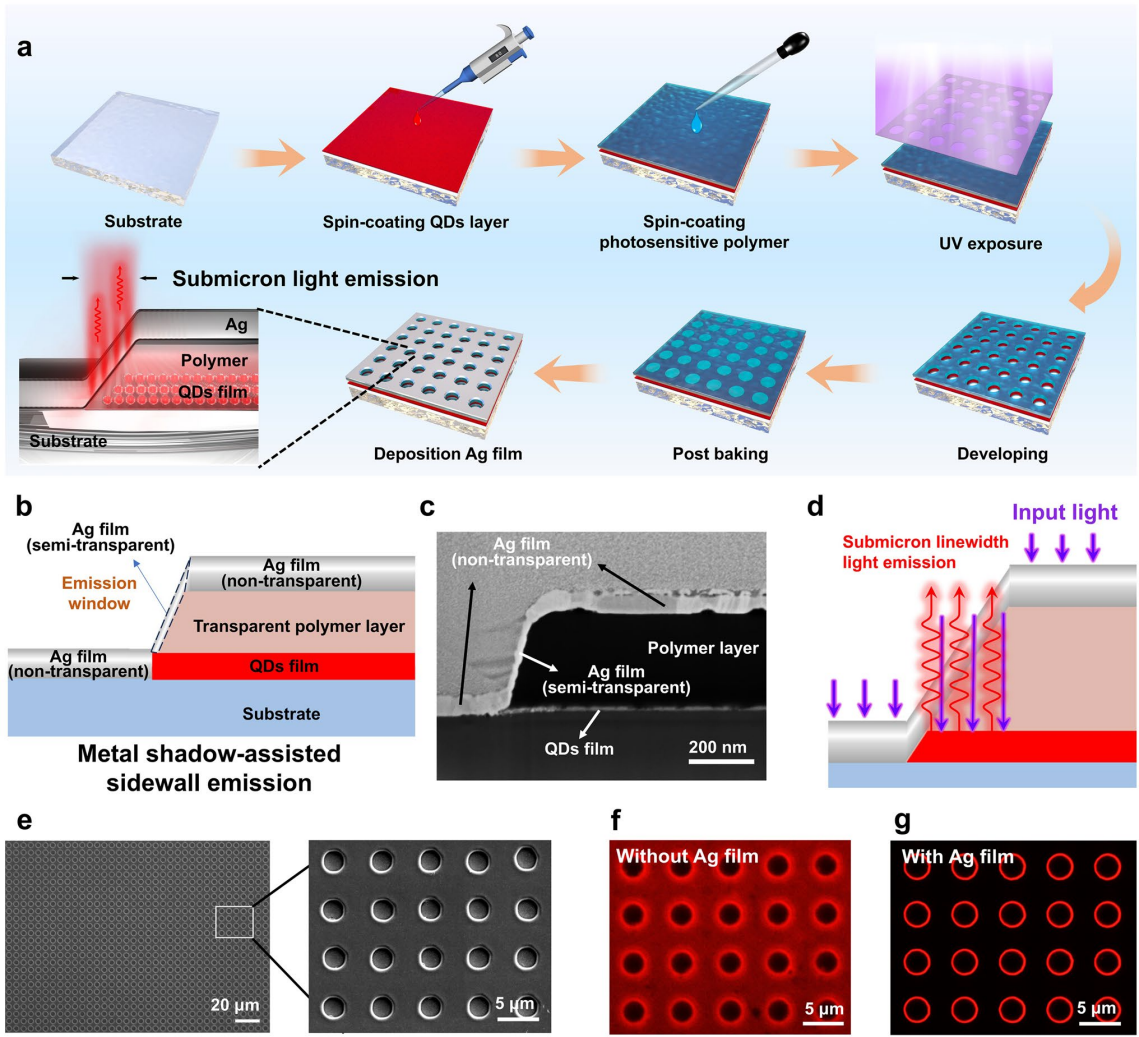
Design of the SASE devices. **a** Fabrication processes of the SASE devices. **b** Design and schematic of the SASE. **c** Cross-sectional SEM image of the SASE. **d** Operation mechanism of the SASE. **e** Microscopy image of surface morphology of a pattern array. The inset: the SEM image of the pattern array. **f**, **g** Fluorescence microscopy image of pattern array without Ag film and with Ag film

This work employs an exceedingly simple process to fabricate the SASE device to achieve submicron linewidths in light source (Fig. 1a). Firstly, one layer of colloidal QDs (~ 40 nm-thick) and one layer of PP (~ 600 nm-thick) are sequentially spin-coated on a cleaned substrate. Then, the substrate is exposed to UV light using a photomask. After the exposure, the exposed photosensitive polymer (PP) is removed, and shaped features are patterned on the glass substrate. Finally, a thin film of Ag is deposited on the patterned glass substrate using thermal evaporation. The fluorescence image of the red ring can be obtained by using UV light to excite the device.

The design schematic of SASE is illustrated in Fig. 1b. The fundamental constituents of the SASE device encompass: (1) A QDs film, which serves as a photoluminescent material and functions as the light source; (2) A semitransparent Ag film, which enables incident light to enter the QD film and acts as an emission window for the emitted light; (3) A non-transparent Ag film, which ensures emission of output light solely from the emission window; (4) Polymer layer, acting as an optical waveguide to direct incident light toward the QDs layer and subsequently guiding excited emission light toward the emission window. Figure 1c shows the cross-sectional SEM image of a fabricated SASE device.

The operational mechanism of the SASE device, which generates submicron linewidth, is illustrated in Fig. 1d. The QD films are excited by incident light and subsequently emit red light. Because of the semitransparence of Ag film in the sidewall of PP, the excited red light could only be emitted from the emission window. The lateral projection size of the sidewall is limited to a few hundred nanometers, leading to the formation of light sources with submicron linewidth. According to the designed operation mechanism, to achieve submicron linewidth of the emitted light, precise control over the horizontal projection size of the emission window is crucial. In this study, the sidewall effect inherent in traditional UV lithography is ingeniously leveraged. This effect introduces a slight tilt angle at the edge of PP, resulting in a significantly reduced horizontal projection size compared to micrometer-scale dimensions. Furthermore, this sidewall feature facilitates the simultaneous fabrication of non-transparent and semitransparent metal layers through a streamlined one-step method utilizing metal thermal evaporation.

In the demonstration, the pattern is an array of holes (Fig. 1e). After the exposure, the exposed PP and QD layer are removed, and circle-shaped features are patterned on the substrate. The SEM image shows the surface morphology of array (inset of Fig. 1e). Next, a thin film of Ag is deposited on the patterned substrate using thermal evaporation. The fluorescence image of the substrate can be obtained by using an UV light to excite the device. Figure 1f, g are the fluorescence images before and after the evaporation of the Ag film, respectively. It can be found that the desired submicron scale light output can be clearly observed after the fabrication of the Ag film. As mentioned above, the one-step thermal evaporation process would lead to the formation of a semitransparent metal film on the PP sidewall and a non-transparent metal film on the PP upper surface. The Ag film deposited on the upper surface of PP layer is relative thick and non-transparent, which can prevent light emission from the upper surface.

### Characterization of SASE Device

**Fig. 2:**
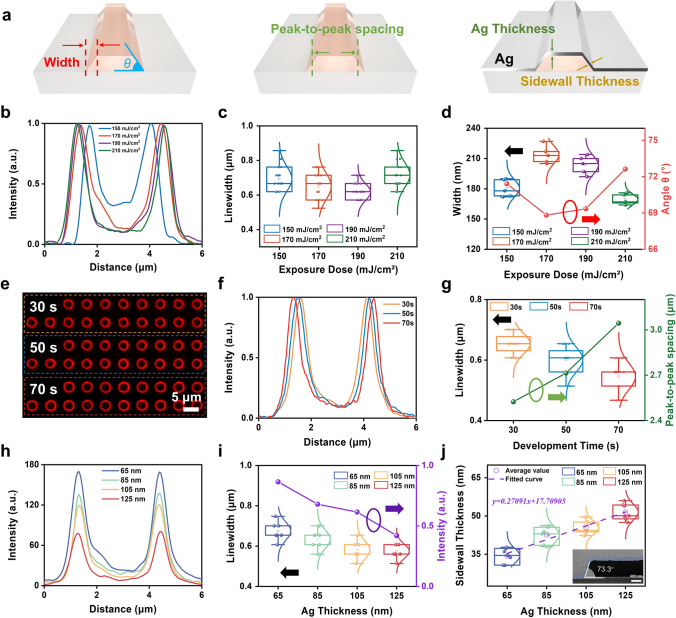
Relationship between factors and linewidth of submicron light sources. **a** A schematic illustration of the defined dependent variables in the preparation process of submicron light sources. **b** Quantitative information, **c** linewidth statistics of submicron light sources, **d** sidewall width statistics and sidewall angles under different exposure doses. **e** Fluorescence microscope images of submicron light sources with development times of 30, 50, and 70 s. **f** Quantitative information, **g** linewidth statistics and the spacing between peaks of submicron light sources under different development time. **h** Quantitative information, **i** linewidth statistics and luminescence intensity, **j** sidewall thickness statistics of submicron light sources under different thickness of Ag film. The inset: the SEM image of the sidewall angles

In the fabrication process of the SASE device, UV light exposure dosage, developing time, and metal thickness play crucial roles. Therefore, the influence of these three factors on submicron linewidths in this work is analyzed. To better explain the impact of various factors on the submicron light source, the dependent variables, including width, sidewall inclination angle *θ*, peak-to-peak spacing, Ag thickness, and sidewall thickness, are defined during the device fabrication process (Fig. 2a). Firstly, the impact of varying exposure doses on the devices is investigated. The devices are fabricated using four different exposure doses: 150, 170, 190, and 210 mJ cm^−2^, whose SEM images and fluorescent microscope images are presented in Figs. S2 and S3. To acquire visual features, the brightness of fluorescence images from devices with different exposure doses is extracted, as illustrated in Fig. 2b. The hole size of 3 μm in the exposure dose of 150 mJ cm^−2^ does not reach the dimensions specified by the mask, indicating incomplete exposure. This discrepancy can be attributed to an insufficient dosage during the exposure process [34]. The insufficient hole size precisely leads to the diffusion of excited red light from the emission windows toward the center, resulting in incomplete darkness within the hole. The hole with an exposure dose of 170 mJ cm^−2^ has been almost completely exposed as the exposure dose increase. In samples with exposure doses of 190 and 210 mJ cm^−2^, the luminescence at the center position of the hole remains almost unchanged, indicating that the samples have been adequately exposed.

To further investigate the characteristics of submicron light sources, the full width at half-maximum of a peak is defined as the linewidth, and the linewidths of the light sources in the four samples are analyzed, as illustrated in Fig. 2c. The linewidth of light sources exhibits a trend of initial narrowing followed by widening as the exposure dose increases, while maintaining the same excitation light intensity. The sample with an exposure dose of 170 mJ cm^−2^ emits red light with the narrowest linewidth. This discrepancy may result from the different susceptibility of PP to varying dosages of radiation [35]. Therefore, the width of the sidewalls and the inclination angle (*θ*) between the sidewalls and the horizontal plane are measured based on SEM images of sample cross sections, as shown in Fig. 2d. The sidewall width exhibits an initial increase followed by a subsequent decrease, in contrast to the variation trend of inclination angle *θ* that initially decreases and then increases. However, the observed trend of inclination angle *θ* is consistent with the variation in linewidths of light sources. A comprehensive elucidation of the underlying factors contributing to this phenomenon will be provided in the subsequent discourse.

It should be noted that the sidewall width in the SEM images (approximately 200 nm) appears significantly smaller than the recorded linewidth in the PL images (approximately 600 nm). The possible reasons are: (1) The linewidth of the submicron light source has approached the diffraction limit of the optical microscope, resulting in a broadening of the emission linewidth during measuring. (2) Due to the existence of the sidewall tilt angle, the emitted light is prone to diffusion and does not have a high directivity. These two factors are the reasons why the linewidth in the PL image is significantly larger than the sidewall width in the SEM image.

In the fabrication of submicron light sources, the developing time is also imperative. The impact of development time on SASE devices is investigated. It is observed that this developing process not only eliminates unwanted PP regions (Fig. S4a), but also eradicates QDs beneath that region (Fig. S4b). The energy dispersive spectrometer (EDS) results indicate the absence of a discernible distribution pattern for Cd and Se (Fig. S4c). It can be noted that the area after developing process no longer contains any quantum dots. It is widely acknowledged that the developing time constitutes a crucial parameter in the patterning process. The three samples are prepared with varying developing times, specifically 30, 50, and 70 s, and the morphology of fabricated patterned arrays has a dynamic transition as the developing time increases (Fig. S5). The duration of the developing process is considered to exert a substantial influence on the diameters of the holes. After thermal evaporation of Ag film onto samples with different developing times, the submicron light sources are shown in Fig. 2e. The fluorescence images of submicron light sources are processed using the image analysis software, allowing for the precise acquisition of luminance information from each pixel. The extracted luminance information of submicron light sources with different developing times is shown in Fig. 2f. It should be noted that although this fluorescence microscope using the image analysis software cannot measure absolute brightness, it can effectively capture relative brightness variations. Traditional luminance meters are incapable of accurately resolving subtle structural changes in submicron light sources. Therefore, using fluorescence microscopy to rapidly analyze the influence of process parameters on submicron light sources is valuable. Figure 2g demonstrates a gradual reduction in the linewidth of the submicron light source as the development time increases. This observation suggests that an extended developing time leads to narrower sidewalls, consequently resulting in a decreased linewidth of the submicron light source. Additionally, the spacing between peaks for three samples is extracted in Fig. 2g. The results demonstrate a positive correlation between development time and peak separation, providing further evidence that an increased development time leads to an enlarged diameter of the holes.

The linewidth of light sources is not only influenced by the exposure dose and developing time, but also affected by the thickness of metal film. To ensure experimental consistency, a patterned substrate is divided into four parts after photolithography, minimizing potential experimental errors associated with photolithography techniques. Four samples with Ag film thickness of 65, 85, 105, and 125 nm are fabricated by thermal evaporation. The SEM images reveal consistent patterns and the uniform surface morphology across all four samples (Fig. S6). The incident light intensity is kept constant to acquire fluorescence microscopy images of samples with different Ag film thicknesses, further improving the accuracy of the experiment (Fig. S7). The luminescence of light sources gradually decreases as the thickness of the Ag film increases. Focused ion beam processing and SEM are used to characterize the cross section of the patterned PP/Ag film on four samples (Fig. S8). Similarly, the luminance information of submicron light sources is extracted from the fluorescence microscope images of the four samples, as shown in Fig. 2h. Figure 2i illustrates the variation in emission linewidth and emission intensity of samples with different Ag thicknesses. The observation reveals a gradual decrease in both linewidth and intensity as the thickness of the metal increases. As the thickness of the metal increases, the emission of excited light from the emission window becomes progressively hindered. The sidewall thickness exhibits a positive correlation with the thickness of Ag layer (Fig. 2j), which is considered the primary factor contributing to the observed variations in emission linewidth and intensity. A linear fit is performed on the average sidewall thickness of four samples with different metal thicknesses, yielding a slope of 0.27091 for the fitted line. Furthermore, the inclination angle of the sidewall is calculated to be 74.2°, which is almost identical to the measured value of 73.4° (inset of Fig. 2j), indicating that sidewall thickness is not only influenced by metal thickness but also related to sidewall angle.

### Mechanism of SASE Device

**Fig. 3:**
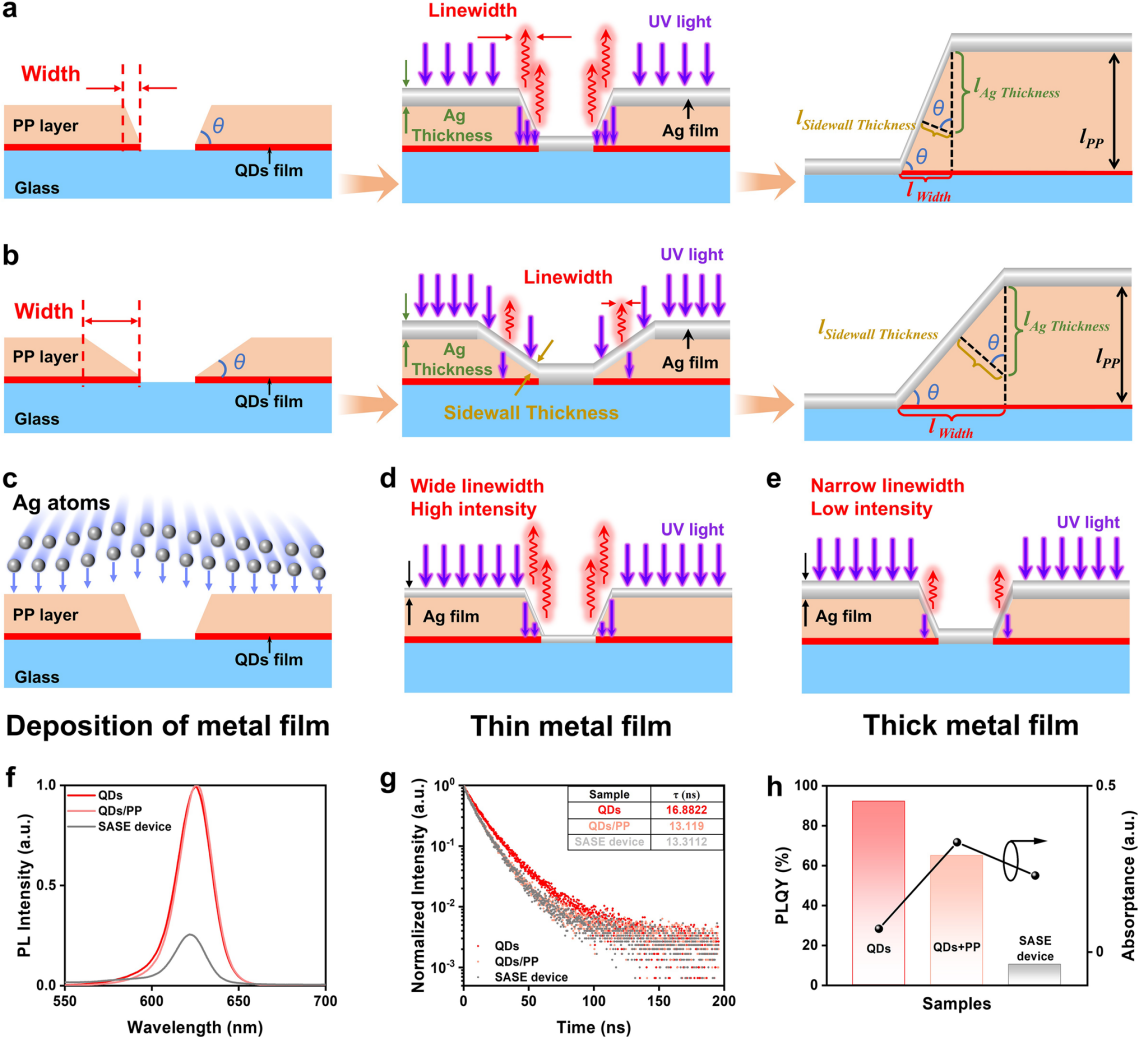
Operation mechanism of the SASE devices. **a** Schematic of the mechanism for the formation of wider linewidths when *θ* is large. **b** Schematic of the mechanism for the formation of narrower linewidths when *θ* is small. **c** Schematic of the deposition of Ag film on PP layer. **d** Schematic of increased light emission from the SASE device with a thin Ag film. **e** Schematic of reduced light emission from the SASE device with a thick Ag film. **f** PL spectra, **g** PL decay curves, and **h** PLQY data and absorptance of QDs, QDs/PP, and the SASE device (Fig. S10)

The experimental results indicate that the device is primarily fabricated through two key processes: photolithography and the deposition of Ag film. Notably, the photolithography process predominantly affects the width (*l*_Width_) and angle (*θ*) of the sidewall. To better analyze how device parameters influence performance of submicron light sources, a mathematical model of the SASE device is established. When the thickness of the PP is defined as *l*_PP_, the width and angle follow the relationship *l*_PP_ = *l*_Width_∙tan*θ*. When maintaining a constant PP thickness, a narrower sidewall width indicates an increased value for angle* θ*, as shown in Fig. 3a. Furthermore, the deposition of the Ag film directly determines the thickness of both semitransparent Ag film (*l*_Sidewall Thickness_) and non-transparent Ag film (*l*_Ag Thickness_) in the SASE device, which is a key factor influencing the PL intensity and linewidth of the submicron light source. Based on the photolithography process, the thickness of the semitransparent Ag film follows the relationship *l*_Sidewall Thickness_ = *l*_Ag Thickness_∙cos*θ*, where *l*_Ag Thickness_ is determined by the thermal evaporation process, and *θ* is governed by the photolithography process. As the optical emission window, the semitransparent Ag film shows a negative correlation between its thickness and the optical characteristics of the submicron light source when the incident light intensity remains constant (corresponding to Fig. 2i, j). Therefore, these device parameters play a crucial role in optimizing the performance of the device.

As the angle *θ* of the device increases, the Ag film deposited in the direction perpendicular to the sidewall, resulting in a smaller value of *l*_Sidewall Thickness_. More light penetrates into the devices through the sidewall to excite QDs for luminescence. Moreover, there is an enhanced probability for excited red-light emission from this specific direction, leading to a broader emission linewidth. Conversely, a smaller *θ* angle leads to an increased *l*_Sidewall Thickness_, thereby reducing the optical transmission capability and the tendency for light emission excitation, ultimately resulting in a narrower emission linewidth, as illustrated in Fig. 3b. The mechanism elucidates the underlying cause for the negative correlation between luminescence linewidth and sidewall width when investigating the effect of exposure dose on submicron light sources.

Furthermore, during the Ag film deposition process (Fig. 3c), the sidewall thickness follows the equation *l*_Sidewall Thickness_ = *l*_Ag Thickness_∙cos*θ*. Under a constant *θ* angle, devices with a smaller *l*_Ag Thickness_ generate a broader emission linewidth and a higher-intensity submicron light source when exposed to excitation light of the same intensity (Fig. 3d). This is because thinner deposited Ag films result in a smaller *l*_Sidewall Thickness_ value, allowing greater light emission. In contrast, when the Ag film is thicker, the *l*_Sidewall Thickness_ value increases, reducing the emitted light and producing submicron light sources with narrower linewidths and lower intensity (Fig. 3e).

As a light-emitting device, the optical performance of the SASE device is of significance, particularly in terms of PL and PLQY. It is worth noting that the optical properties of the SASE device are primarily determined by the intrinsic properties of the materials used. The analysis of process variables in SASE device fabrication, including exposure time, development time, and metal film thickness, aims to optimize structural parameters and examine their impact on the linewidth of the SASE device, which is a key parameter in submicron light sources. Therefore, without altering the materials used, optimizing structural parameters has a minimal impact on the optical performance of the device.

The PL spectra of QDs films and QDs/PP films indicate that under identical excitation conditions, the PL intensity of the QDs/PP film remains nearly unchanged compared to that of the QDs film, with only a slight redshift (measurement method shown in Fig. S9a, b). This redshift may be attributed to red-emitting dyes in the PP being excited by incident light, leading to longer-wavelength emission. The SASE device exhibits a relatively low PL intensity due to the opaque Ag film, which blocks the emission from the QDs (Fig. 3f). Moreover, the time-resolved PL reveals that the PL decay of the QDs film is significantly slower than that of the QDs/PP film, as shown in Fig. 3g. This suggests the presence of additional PL decay channels in the QDs/PP film, indicating a major influence of PP on the QDs film. The degradation of QDs caused by PP primarily arises from two factors: Firstly, the organic solvents in the PP can destroy the surface ligands of QDs, introducing defects that degrade their performance. Secondly, the photosensitizers in the PP may generate free radicals, leading to the chemical degradation of QDs, damaging their surface structure, and ultimately reducing their PL performance. The PL decay curves of the SASE device and the QDs/PP films are nearly identical, which can be attributed to the protective effect of the PP, preventing damage to the QDs during the Ag deposition process. As for the PLQY, this parameter is determined not only by the number of emitted photons but also by the absorbance of the sample (measurement method shown in Fig. S9c, d). A stronger absorption might lead to a low PLQY. As for the QDs/PP film, the absorption of UV by PP film would increase the total absorbance of the sample (Fig. 3h).Therefore, although the PL intensity of the QDs film and the QDs/PP film is almost the same in the PL spectrum measurements, the PLQY of the QDs/PP film is lower due to the strong UV absorption of PP. Additionally, the lower PL intensity, combined with the absorption of both excitation light and QDs emission by the Ag film, further reduces the PLQY of the SASE device to approximately 10% (Fig. 3h). We have to acknowledge that the light is emitted only from the lateral emission window, resulting in a lower utilization efficiency of QDs PL. However, trading this efficiency to achieve a submicron light source is a worthwhile compromise. This is because the proposed ultra-simple method enables the fabrication of submicron light sources using only conventional exposure equipment and a simple process, eliminating the need for advanced equipment and complex fabrication processes.

### Scalability of SASE Device

**Fig. 4:**
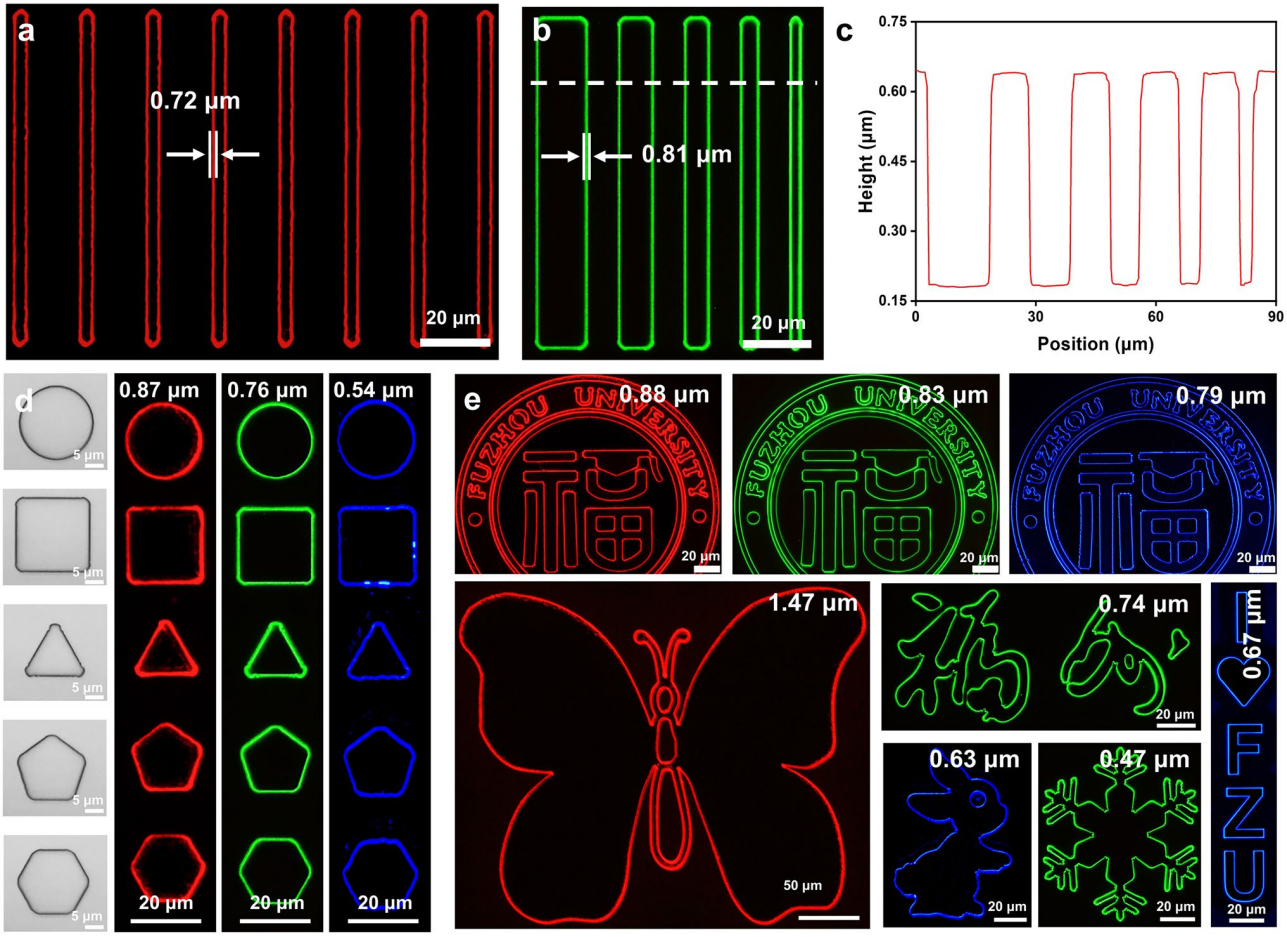
Application and characterization of submicron light source. **a** Red submicron light source with a linewidth of 0.72 µm. **b** Green submicron light source with a linewidth of 0.81 µm. **c** Height profile of pattern array corresponding to the white dashed line in Fig. 4b. **d** Optical images of fundamental geometric shapes and their corresponding red, green, and blue submicron light sources. **e** Fluorescence images of submicron light sources with various complex patterns, including the university logo (red, green and blue), animals (red and blue), Chinese characters (green), symbol (green), and letters (blue). (Colour figure online)

Through the utilization of this remarkably straightforward technique, a series of QD patterns with submicron linewidths are designed using red, green, and blue QDs. The fluorescence microscope images in Fig. 4a demonstrate the fabrication of a series of equidistant red submicron light sources with a width of 0.72 μm and a spacing of 2.4 μm. Subsequently, submicron light sources with varying inter-stripe spacing are fabricated in Fig. 4b. The height profiles of pattern arrays characterized by using a 3D measure laser microscope demonstrate clearly defined and sharp edges, as shown in Fig. 4c. In addition to straight lines and circles, submicron light sources can be formed into some basic geometric shapes according to the trajectory of sidewalls. Fig. 4d illustrates the microscope images and fluorescent images of some basic geometric shapes, demonstrating the potential of the method to create arbitrary luminescent patterns. Therefore, examples of the school logos, animals, Chinese characters, letters, and symbols composed of submicron light sources are demonstrated in Fig. 4e. Furthermore, using the SASE method proposed in this work, we successfully demonstrate that four different PPs can be applied to submicron light source fabrication, confirming the general applicability of this method, as shown in Fig. S11. The use of negative PPs poses challenges due to the formation of "inverted trapezoid" sidewalls. Thus, overcoming these challenges may require structural modifications or further optimization of process parameters.

It is worth noting that the minimum achievable spacing of submicron light sources is influenced by multiple factors: (1) Resolution of the photolithography process. The photolithography system used in this study achieves a minimum pattern spacing of 2 μm. To further decrease the pattern spacing, it is essential to enhance lithographic resolution by employing shorter-wavelength exposure sources and optical systems with higher numerical apertures. (2) Thickness of the PP film. The use of a thinner PP film is more conducive to reducing the pattern spacing. Employing thick PP films can introduce significant dissolution gradients during development, leading to pattern collapse at smaller spacings. Moreover, internal light scattering within thicker PP films can blur the exposure boundaries, further constraining the minimum achievable spacing. Therefore, using the thin PP film and overcoming lithographic resolution limits are essential for fabricating submicron light sources with smaller spacing.

**Fig. 5:**
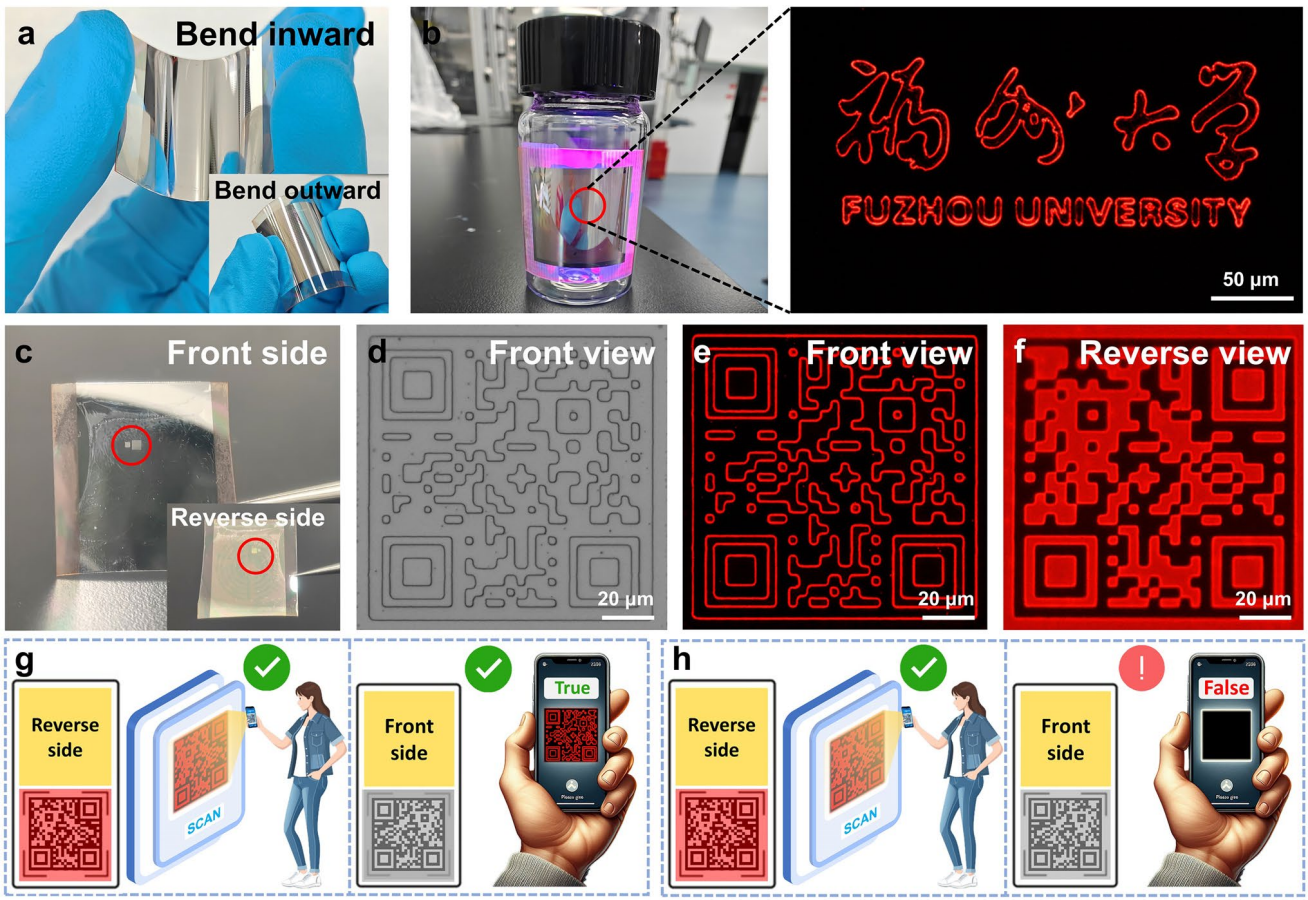
**a** Photograph of a flexible device bents inward and bents outward. **b** Flexible device adhered to an 18 mm diameter bottle. Inset: A zoomed-in submicron light source. **c** Front-side and reverse-side photographs of a flexible device. **d** QR code pattern and **e** submicron light source corresponding to the red circled area of the front side in Fig. 5c. **f** Fluorescence image of QR code corresponding to the red circled area of the reverse side in Fig. 5c Inset. **g** Retrieve valuable information from the reverse side and observe the anti-counterfeiting stripes on the front side. **h** Retrieve valuable information from the reverse side, while the front side lacks anti-counterfeiting stripes. (Colour figure online)

To further demonstrate application scalability, fluorescent patterns are successfully fabricated on a polyethylene naphthalene-2,6-dicarboxylate (PEN) flexible substrate. Figure 5a demonstrates that the fluorescent patterns fabricated on a PEN substrate exhibit both inward-bending and outward-folding capabilities with excellent conformability to curved surfaces. The optical photograph exhibits a fluorescent pattern excited by UV light, and the PEN substrate adheres to a glass bottle with a diameter measuring 18 mm, as shown in Fig. 5b. The enlarged fluorescent image remains as clear and undistorted as those on the glass substrate (inset of Fig. 5b), which reveals the robustness of our approach to fabricate the luminescent stripes with submicron line widths.

### Application of SASE Devices in Optical Anti-Counterfeiting

Currently, quick response (QR) code-based authentication is a commonly used method for verifying authenticity. Although this method raises the technical threshold for counterfeiting and helps mitigate the prevalence of counterfeit products, it is not entirely reliable. QR codes remain vulnerable to forgery through the replication of fraudulent websites. Therefore, further advancements in optical anti-counterfeiting technologies are necessary to enhance security and reliability. The perfect integration of our proposed method with flexible substrates has made it possible for the method to be applied in optical anti-counterfeiting. Flexible devices for application in optical anti-counterfeiting have been fabricated, as shown in Fig. 5c. The front side of the device exhibits an Ag film that has been deposited through evaporation onto the surface of the PP. The inset in Fig. 5c presents a photograph of the device after it has been flipped, providing a reverse view. Figure 5d shows the QR code pattern of the anti-counterfeiting mark under the microscope. When the surface is illuminated by a UV light, only the outline of the QR code is visible, as shown in Fig. 5e. The submicron linewidth outline fabricated by our method is highly challenging to forgery and counterfeiting, making them a crucial feature for dual-layer anti-counterfeiting. Moreover, when the device is illuminated from the back with a UV lamp, the true QR code pattern becomes visible under the microscope, allowing for the retrieval of useful information, as shown in Fig. 5f. In this process, the submicron light source plays a crucial role as an important feature for dual-layer optical anti-counterfeiting. Compared to conventional QR code-based anti-counterfeiting techniques, the proposed SASE method enables the fabrication of submicron light sources for dual-layer anti-counterfeiting using simple fabrication processes and conventional equipment. This simple method significantly enhances security and offers substantial advantages in the field of optical anti-counterfeiting.

The application of submicron light sources in optical anti-counterfeiting is successfully demonstrated. As shown in Fig. 5g, the scanning of the QR code on the reverse side provides valuable information (left view), and the detection of a submicron light source of QR code pattern from the front side confirms its authenticity (right view). If the QR code can be scanned to retrieve information (left view), but no submicron light source corresponding to the QR code is detected on the reverse side of the identification (right view), it can be inferred that the item is counterfeit, as illustrated in Fig. 5h. Therefore, the submicron light source fabrication method proposed in this work shows promise for applications in the field of optical anti-counterfeiting.

## Conclusion

The article proposes the SASE for fabricating submicron-sized photoluminescent light sources using conventional lithography techniques. It circumvents the need for complex micro-nano fabrication technologies and advanced processing equipment, achieving submicron linewidth photoluminescent light sources by exploiting the sidewall effect during the photolithography process. The experimental results demonstrate that precise control over the linewidth of submicron light sources can be achieved by adjusting process parameters, including exposure time, development time, and metal film thickness. The formation mechanism of the submicron light sources is proposed, with the sidewall angle of the polymer being the primary factor. Within a certain range, there is a positive correlation between the linewidth of the submicron light sources and the sidewall angle. When depositing metal films with identical thicknesses, a smaller sidewall angle results in a reduced linewidth of submicron light sources. Furthermore, the potential of the SASE method to fabricate arbitrary patterns is successfully demonstrated. The integration of a flexible substrate with a submicron light source effectively demonstrates its potential for optical anti-counterfeiting applications. The results of this study indicate that the SASE offers a new perspective for the research and application of micro light sources.

## Supplementary Information

Below is the link to the electronic supplementary material.

Supplementary file1 (DOCX 7238 kb)
