# Supplementary material for: Shadow-Assisted Sidewall Emission for Achieving Submicron Linewidth Light Source by Using Normal UV Photolithography
Source: Nanomicro Lett. 2025 Apr 22;17:228. doi: 10.1007/s40820-025-01737-w (PMC12014993; doi:10.1007/s40820-025-01737-w)
Supplement: Supplementary file 1 — Supplementary file1 (DOCX 7238 kb) [file 40820_2025_1737_MOESM1_ESM.docx]

Supporting Information for

**Shadow-Assisted** **Sidewall Emission for Achieving** **Submicron Linewidth Light Source by Using Normal UV Photolithography**

Junlong Li^1, ‡^, Yanmin Guo^1, ‡^, Kun Wang^1, ‡^, Wei Huang^1^, Hao Su^1^, Wenhao Li^1^, Xiongtu Zhou^1,2^, Yongai Zhang^1,2^, Tailiang Guo^1,2^, and Chaoxing Wu ^1, 2,^ *

^1^College of Physics and Information Engineering, Fuzhou University, Fuzhou 350108, P. R. China

^2^Fujian Science & Technology Innovation Laboratory for Optoelectronic Information of China, Fuzhou 350108, P. R. China

^‡^ Junlong Li, Yanmin Guo, and Kun Wang contributed equally to this work.

*Corresponding author. E-mail: [chaoxing_wu@fzu.edu.cn](mailto:chaoxing_wu@fzu.edu.cn) (Chaoxing Wu)

**Supplementary Figures**

**
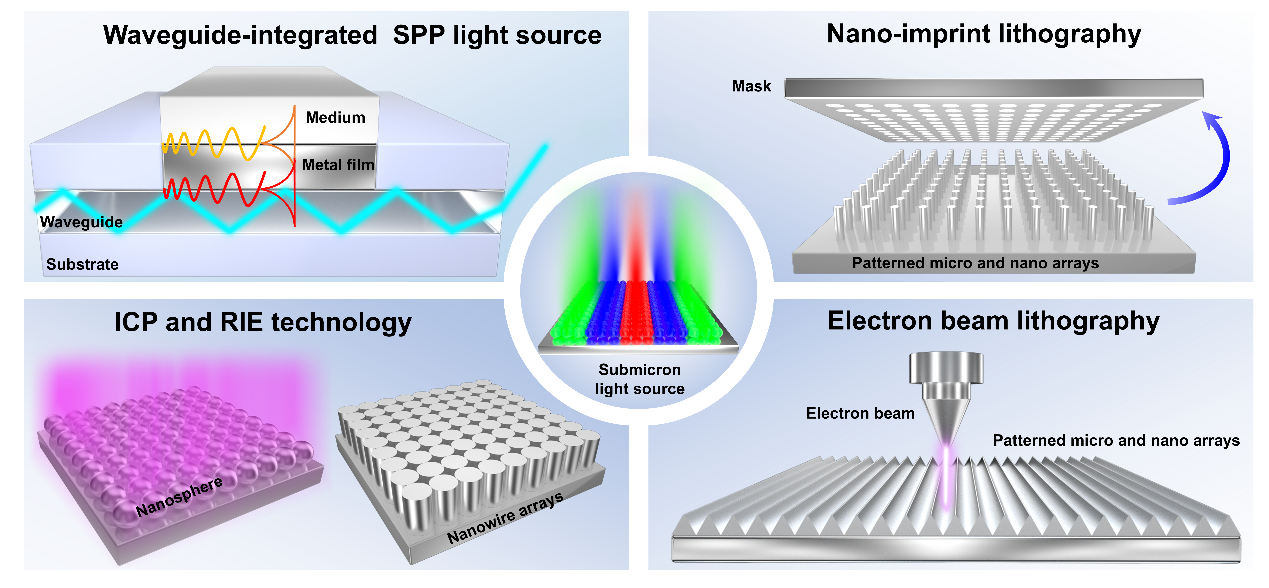
Fig. S1** Fabrication strategies for submicron light sources. Advanced equipment such as nanoimprinting and electron beam lithography machines, and complex fabrication processes like plasma etching technology and surface plasmon polaritons are necessary for fabricating submicron light sources.


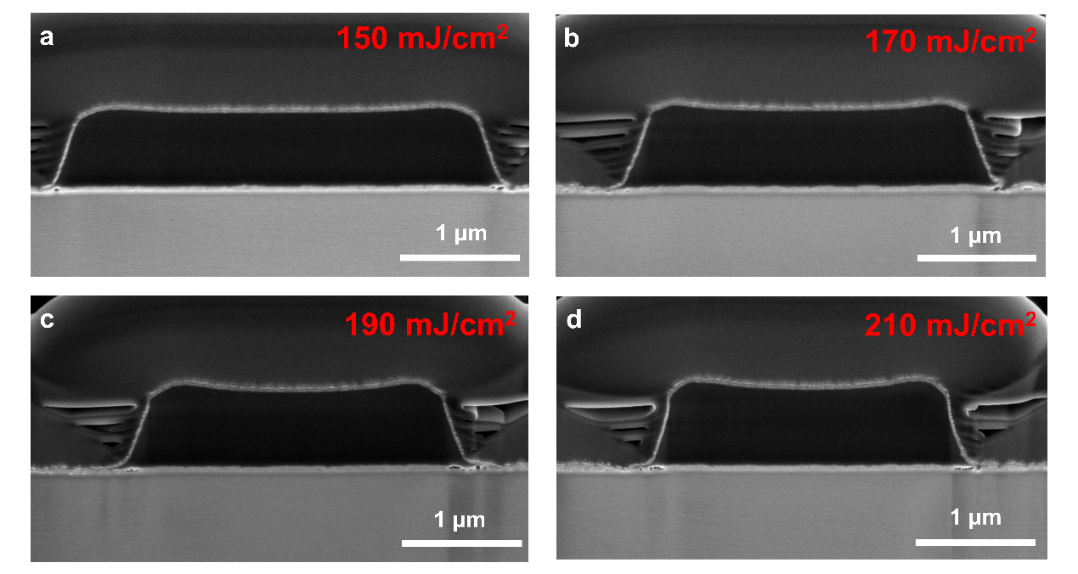


**Fig. S2** Relationship between exposure dose and surface morphology of the device. Cross-section SEM images of pattern array with exposure dose of **a** 150 mJ/cm^2^, **b** 170 mJ/cm^2^, **c** 190 mJ/cm^2^ and **d** 210 mJ/cm^2^


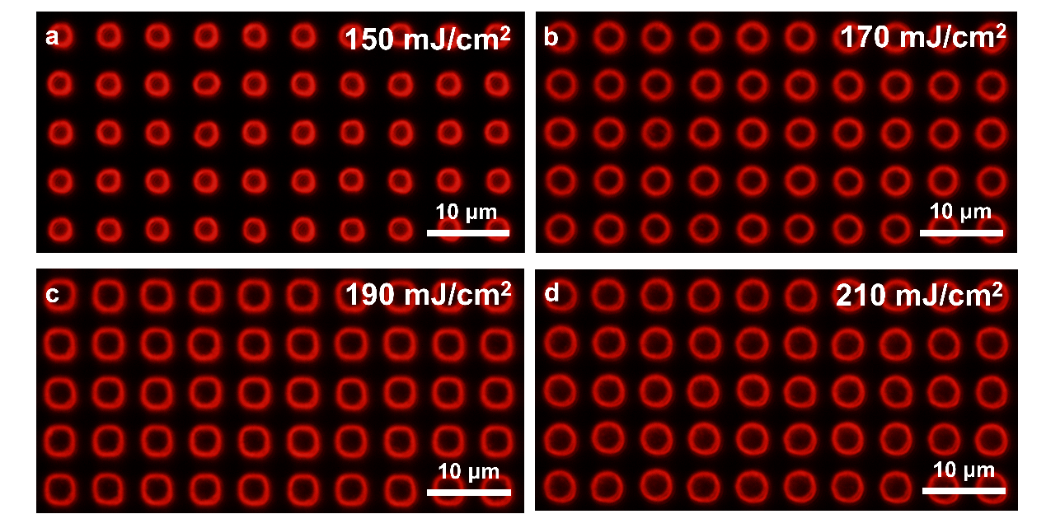


**Fig. S3** Relationship between exposure dose and linewidth of submicron light sources. Fluorescence microscope images of submicron light sources with exposure dose of **a** 150 mJ/cm^2^, **b** 170 mJ/cm^2^, **c** 190 mJ/cm^2^ and **d** 210 mJ/cm^2^


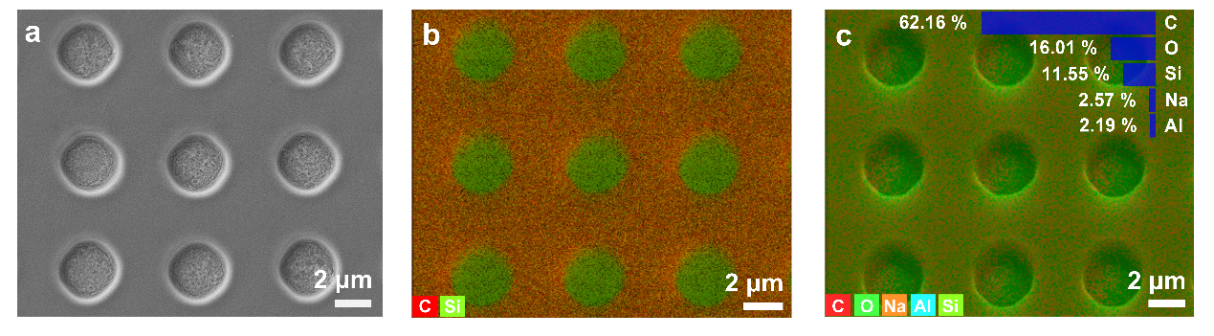


**Fig. S4** Chemical element analysis of the MASE device. **a** SEM image and **b** energy dispersive spectrometer mapping of pattern array. **c** Energy dispersive spectrometer mapping of the primary chemical elements in the SASE device. The results indicate that the absence of a discernible distribution pattern for Cd and Se. It can be noted that the area after developing process no longer contains any quantum dots


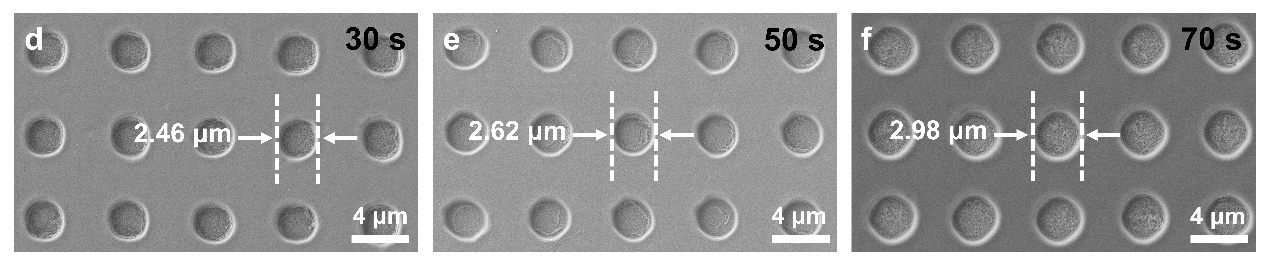


**Fig. S5** Relationship between developing time and surface morphology of the device. The SEM images of morphologic transition of pattern array with development time of **a** 30 s, **b** 50 s and **c** 70 s


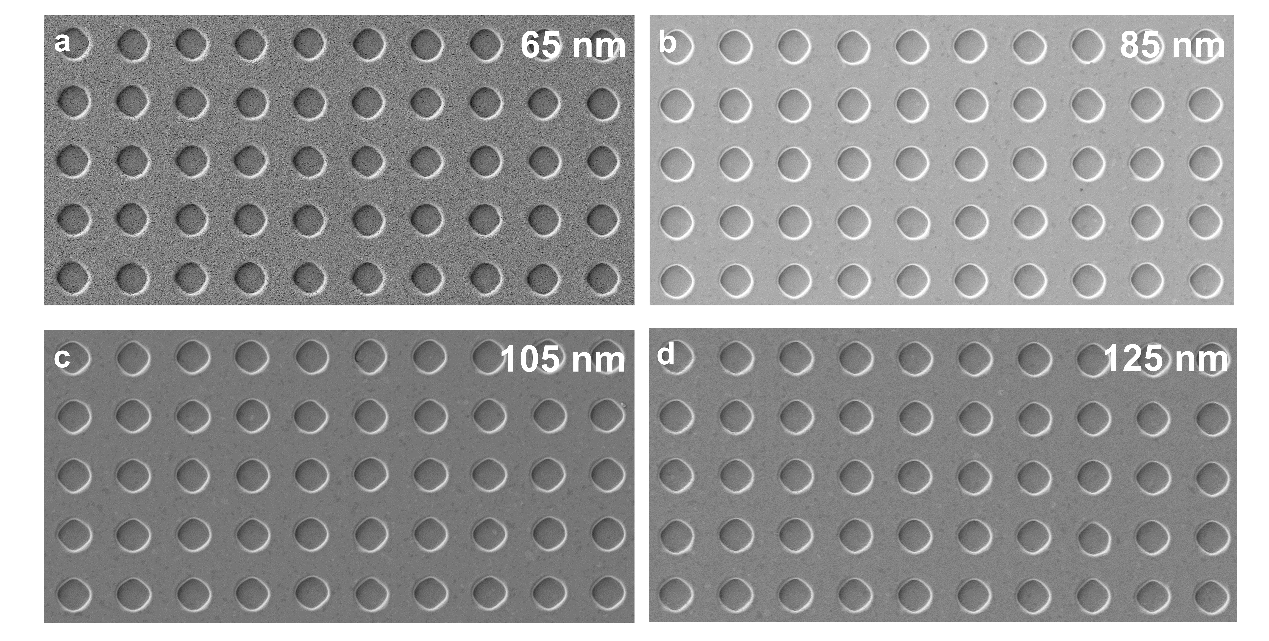


**Fig. S6** Surface morphology of SASE devices with different metal film thicknesses. SEM images of pattern array with **a** 65 nm, **b** 85 nm, **c** 105 nm, and **d** 125 nm Ag film


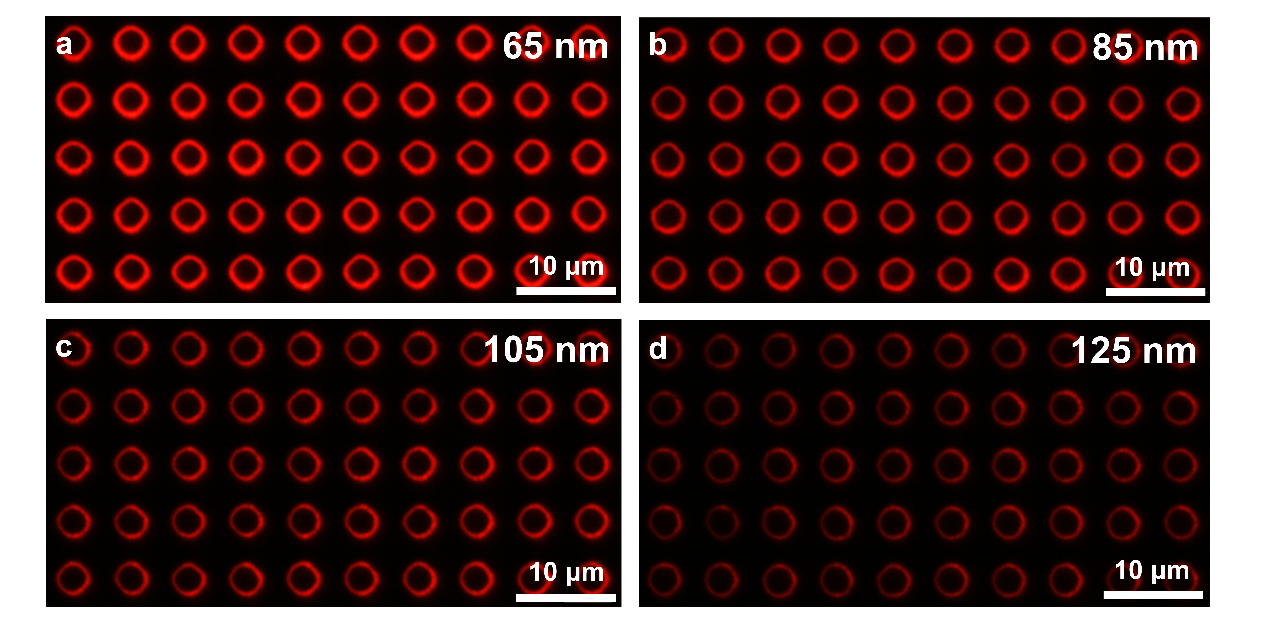


**Fig. S7** Relationship between metal film thickness and submicron light source. Fluorescence microscope images of submicron light sources with **a** 65 nm, **b** 85 nm, **c** 105 nm, and **d** 125 nm Ag film


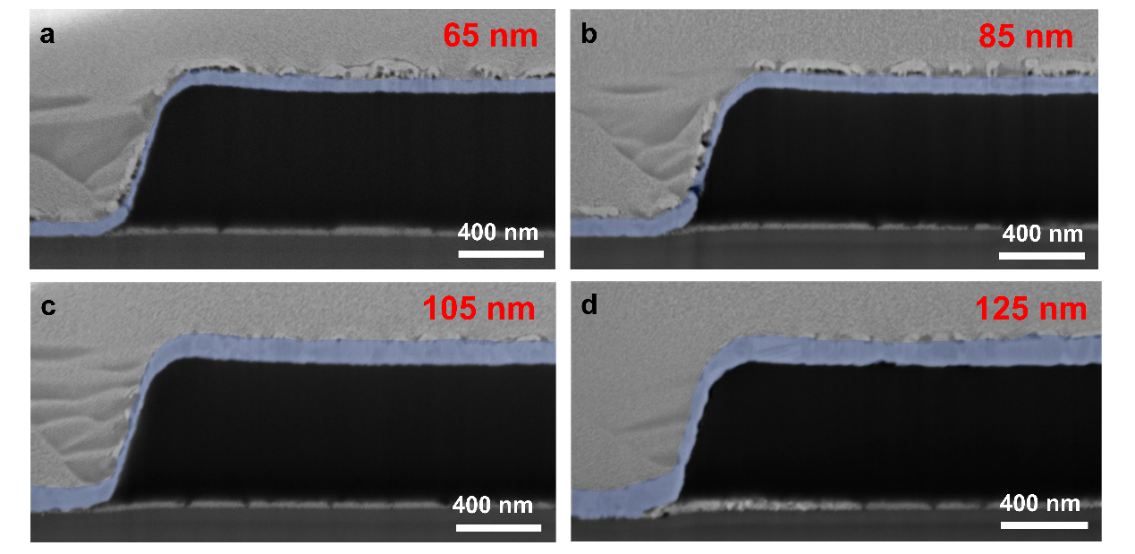


**Fig. S8** Cross-section of SASE devices with different metal film thicknesses. Cross-section SEM images of submicron light sources with **a** 65 nm, **b** 85 nm, **c** 105 nm, and **d** 125 nm Ag film


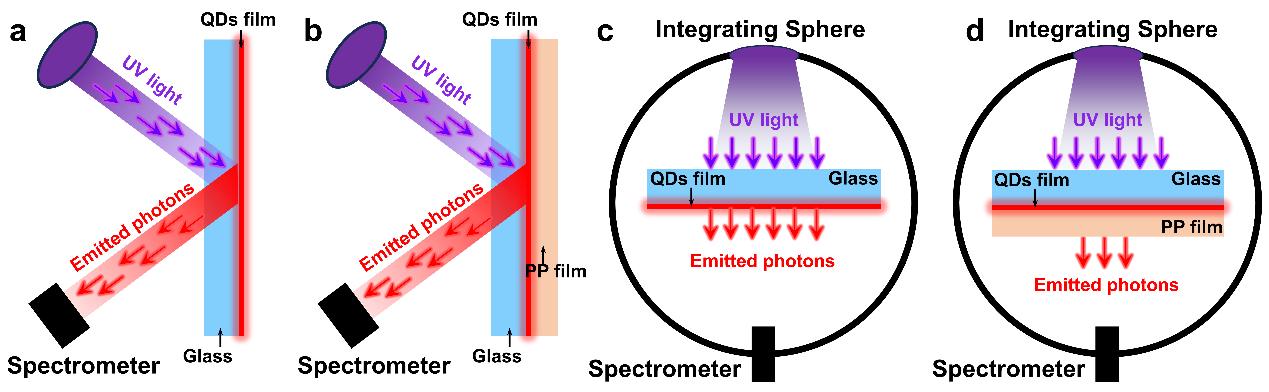


**Fig. S9** Schematic diagrams of the PL and PLQY measurement. PL measurement method for **a** the QDs film and **b** QDs/PP film; PLQY measurement method for **c** the QDs film and **d** QDs/PP film. When measuring the PL spectra of the samples, the emitted red light is collected from the glass side, and subsequently detected by the spectrometer. When measuring the PLQY, the emitted light is collected within the integrating sphere and subsequently detected by the spectrometer


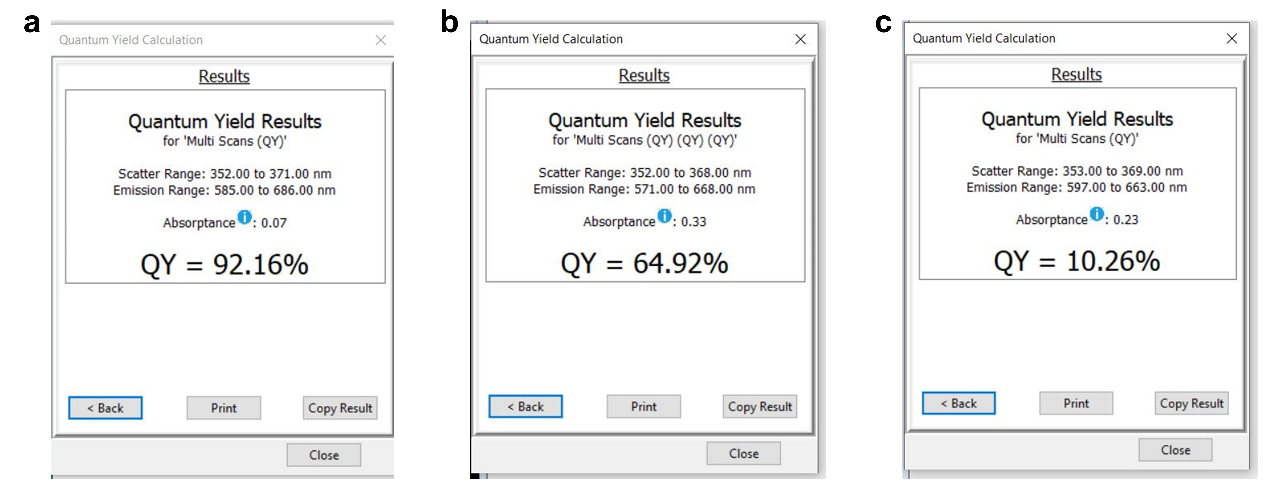


**Fig. S10** PLQY and absorptance of **a** QDs film, **b** photosensitive polymer coated-QDs film, and **c** SASE device


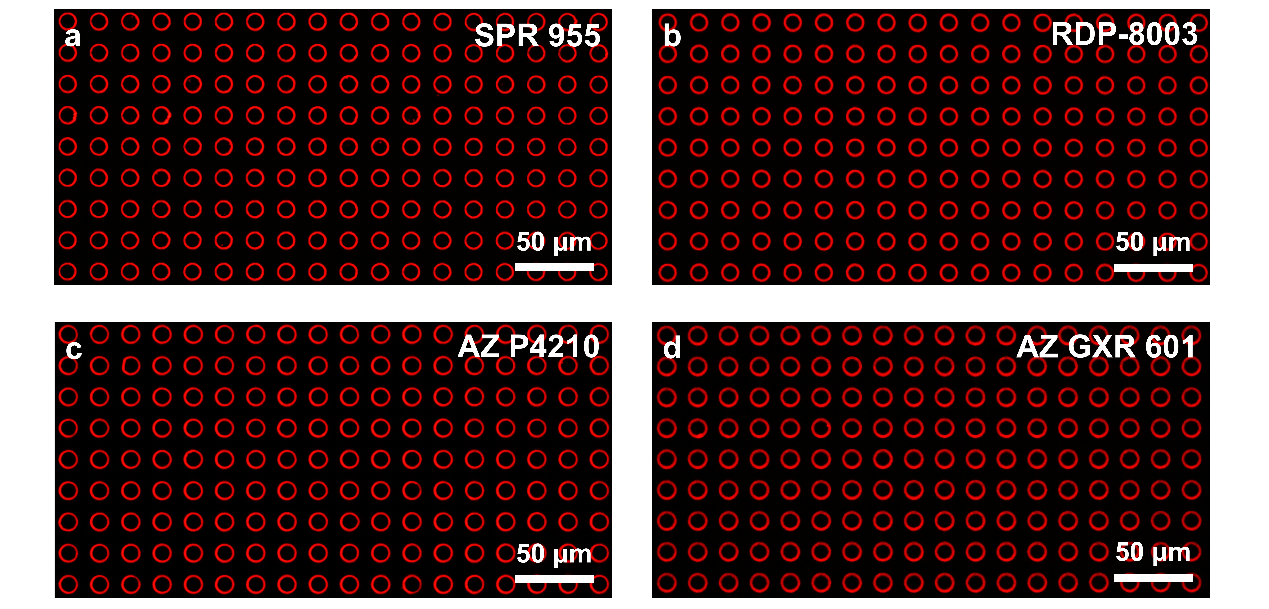


**Fig. S11** The general applicability of the SASE method. The feasibility of the SASE method for submicron light source fabrication is demonstrated using **a** SPR 955, **b** RDP-8003, **c** AZ P4210, and **d** AZ GXR 601 PPs. Submicron light sources can be successfully fabricated using different positive PPs, demonstrating that the SASE method is a generally applicable strategy
